# Clinico-epidemiological nature and antibiotic susceptibility profile of *Acinetobacter* species

**DOI:** 10.12669/pjms.292.3132

**Published:** 2013-04

**Authors:** Shirin Biglari, Alfizah Hanafiah, Ramliza Ramli, Md. Mostafizur Rahman, Tzar Mohd Nizam Khaithir

**Affiliations:** 1 Shirin Biglari, Department of Medical Microbiology and Immunology, University Kebangsaan Malaysia Medical Centre, Kuala Lumpur, Malaysia.; 2 Alfizah Hanafiah, Department of Medical Microbiology and Immunology, University Kebangsaan Malaysia Medical Centre, Kuala Lumpur, Malaysia.; 3 Ramliza Ramli, Department of Medical Microbiology and Immunology, University Kebangsaan Malaysia Medical Centre, Kuala Lumpur, Malaysia.; 4 Md. Mostafizur Rahman, Department of Medical Microbiology and Immunology, University Kebangsaan Malaysia Medical Centre, Kuala Lumpur, Malaysia.; 5 Tzar Mohd Nizam Khaithir, Department of Medical Microbiology and Immunology, University Kebangsaan Malaysia Medical Centre, Kuala Lumpur, Malaysia.

**Keywords:** *Acinetobacter* spp., Antimicrobial susceptibility, Intensive care unit, Polymyxin B

## Abstract

***Objectives:*** *Acinetobacter *spp. has emerged as an important opportunistic pathogen responsible for nosocomial infections in many health-care settings worldwide. The study describes the clinico-epidemiology and antimicrobial susceptibility of *Acinetobacter* spp. in a tertiary health-care institution.

***Methodology***: *Acinetobacter* spp. were isolated from 141 specimens of the patients who reported to Universiti Kebangsaan Medical Centre (UKMMC). The sources of specimens were wound, skin and soft tissue, respiratory and urinary tract from patients in various wards. Clinio-epidemiological features of patients infected with *Acinetobacter* spp. were recorded. Standard bacteriological techniques with API 20NE kits and disk diffusion method were followed for identification and antibiotic sensitivity of the organisms.

***Results:*** One hundred and forty one patients with positive culture for *Acinetobacter* spp. were identified. Soft tissue/wound and respiratory tract were among the commonest sites of *Acinetobacter* spp. isolation. The isolates were most frequently obtained from ICU. All isolates were multi-drug resistant and had a resistance rate of more than 70% to most antibiotics, except polymyxin B.

***Conclusion:*** High prevalence of multi-drug resistance *Acinetobacter *spp. provides essential information on judicious antibiotic selection for empirical therapy in our health-care institution.

## INTRODUCTION

Infections caused by* Acinetobacter* spp. have become a serious concern in many health-care institutions worldwide. Acinetobacter calcoaceticus–baumannii complex has been recognized as one the most common species responsible for nosocomial bacteremia, meningitis, respiratory tract and urinary tract infections.^1^

The prevalence and antimicrobial susceptibility profiling of *Acinetobacter* spp. has been reported in Malaysian hospitals.^2^^,^^3^ However, the result of the studies might not represent our institution.

The aim of this study was to determine, the demographic and clinical profile of *Acinetobacter *spp. The antimicrobial resistance patterns of *Acinetobacter* strains will be assessed.

## METHODOLOGY

***Setting:*** This is a cross-sectional retrospective observational study conducted in a tertiary healthcare facility with 830 beds.

***Bacterial isolates:*** From October 2010 to April 2011, non-duplicate isolates of* Acinetobacter *spp. grown from all clinical specimens of hospitalized patients were analyzed. The sources of isolates included blood, sputum, tracheal aspirate, bronchoalveolar lavage, pus, sterile body fluid and urine. In this study, nosocomial isolate was defined as isolate grown from specimen that was sampled after 48 hours of hospitalization. Non-nosocomial isolate was defined as isolate grown from specimen sampled within 48 hours of hospitalization. Colonizer was defined as isolate that had microscopy smear showing 0 to 1 pus cell/high power field. Isolate showing more than 1 pus cell/high power field was regarded as significant isolate.

***Patient data:*** Medical and demographic data of hospitalized patients with culture-positive *Acinetobacter *spp. were retrieved from patients’ medical records. Data that were recorded include age, gender, ward location, date of hospitalization, transfer and discharge, date of specimen sampling, specimen site, ICU stay and antibiotic usage.

***Laboratory identification:*** Microbiological data were obtained from laboratory records. Bacterial colonies grown on MacConkey plates were identified by its colonial morphology, Gram-staining and oxidase test. Genus identification was performed using conventional biochemical tests. For blood and sterile body fluid specimens, speciation was performed using API 20NE system, based on manufacturer’s instruction (bioMérieux, France).

***Antimicrobial susceptibility test:*** The antimicrobial susceptibility testing was assessed by disk diffusion method, according to the guidelines of Clinical and Laboratory Standards Institute (CLSI).^4^ Antibiotic disks were obtained from Oxoid Ltd. (Basingstoke UK). Tigecycline susceptibility testing was assessed by disk diffusion method, according to the guidelines of The Asia Pacific Clinical Microbiology Working Group Laboratory Manual 2009. Antibiotic disks were obtained from Becton Dickinson (USA). Minimum inhibitory concentrations (MIC) for polymyxin B were determined by E-tests, based on manufacturer’s instructions (AB Biodisk, Solna Sweden). Quality control was performed with the following strains as recommended by the CLSI:* Escherichia coli* ATCC 25922, *Escherichia coli* ATCC 35218 and *Pseudomonas aeruginosa* ATCC 27853. Isolates were tested for susceptibility to sixteen relevant drugs. Multiple-drug resistant (MDR) *Acinetobacter *spp. was defined as resistance to 3 or more classes of antibiotic used to treat *Acinetobacter* infections.

***Statistical analysis:*** Statistical analysis was performed by using Statistical Package for the Social Science for Windows (version 18.0; SPSS Inc, Chicago, IL, USA). Parametric variables were assessed using chi-squared test, as appropriate. A difference was considered statistically significant if the *p-*value < 0.05.

***Ethical considerations:*** The institution’s medical research and ethical committee had approved this study.

## RESULTS

**Fig.1:**
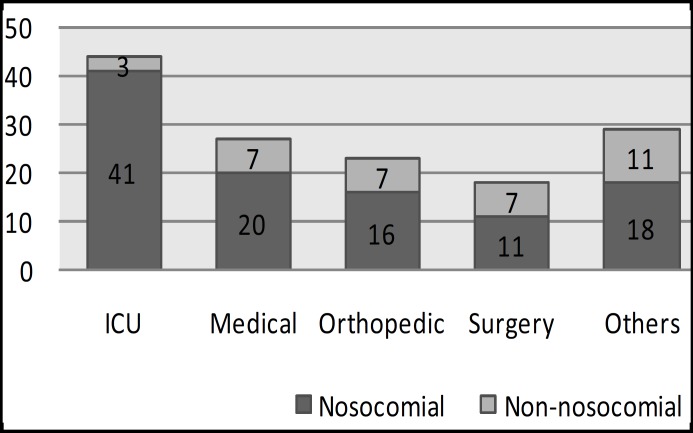
Distribution of *Acinetobacter *spp. Isolates

A total of 141 non-duplicate isolates of* Acinetobacter *spp. grown from all clinical specimens were included in this study. Distribution for the 141 isolates was as shown in Fig.1. The isolates were most frequently derived from ICU, followed by medical, orthopedics and surgery wards. Soft tissue/wound (43.3%) and respiratory tract (31.2%) were among the commonest sites of isolation (Table-I). The isolates were predominantly colonizers (53.2%). Only 66/141 (46.8%) isolates were regarded as significant based on the presence of pus cells in Gram stain smear.

**Table-I:** Distribution of the isolates in relation to specimen site

*Specimen Site*	*No. of Acinetobacter spp. (%)*
Blood Respiratory tract Soft tissue/wound	8 (5.7) 44 (31.2) 61 (43.3)
Sterile body fluid Urinary tract	4 (2.8) 12 (8.5)
Others	12 (8.5)
Total	141 (100)

The demographic characteristics and clinical epidemiology profile of hospitalized patients with culture-positive *Acinetobacter* spp. are shown in Table-II. Bacterial isolates were mostly from males 85 (60.3%). The median age of patients was 54 (IQR 31-66). Overall, 75.2% (106/141) of the isolates were of nosocomial origin. There were 24.8% (35/141) non-nosocomial isolates and 45.6% (17/35) of these strains were from patients whom had previous hospitalization. The duration taken for patients to acquire nosocomial *Acinetobacter* spp. infection/colonization varied from 3 days to 78 days, median duration was 13 days (IQR 7-23).

In this study, 93/141 patients (66.0%) received antibiotics before the isolation of *Acinetobacter* spp. Thirty-seven of them (39.8%) had one course of antibiotic therapy. Thirty out of ninety-three (32.3%) patients had 2 courses of antibiotic therapy and 26/93 (28.0%) patients had three or more courses of antibiotic therapy prior to *Acinetobacter* spp. isolation. The most common antibiotic used was meropenem (22.5%), followed by ceftriaxone (17.6%), piperacillin-tazobactam (13.4%) and amoxicillin-clavulanate (12.0%).

The susceptibility profile of 141 *Acinetobacter* spp. isolates is shown in Table-III. In general, *Acinetobacter *isolates had more than 70% resistance to most antibiotics tested. The rates of more than 70% resistance for antibiotics were ampicillin (95.0%), cefuroxime-parenteral (80.8%), cefotaxime (78.0%), amoxicillin-clavulanate (75.9%), ciprofloxacin (73.8%), ceftazidime (73.0%), meropenem (73.0%), imipenem (72.3%), piperacillin-tazobactam (72.7%), cefepime (73.1%), ampicillin-sulbactam (70.9%) and gentamicin (70.2%). Out of 141 isolates, 79.4% were MDR *Acinetobacter* spp. Twenty-eight isolates were tested for polymyxin B and were 100% sensitive. The MIC90 was 2 mcg/mL. Comparison of the rate of antibiotic resistance between ICU and non-ICU isolates did not show any significant difference (p>0.05).

**Table-II:** Characteristics of the study population (n=141

Age	54 (31-66)
Male	85 (60.3)
Female	56 (39.7)
Nosocomial isolates	106/141(75.2)
ICU	41 (38.6)
Medical	20 (18.9)
Surgery	11 (10.4)
Orthopedics	16 (15.1)
Others	18 (17.0)
Days taken to acquire nosocomial *Acinetobacter*	
Median (Q1-Q3)	13 days (7-23)
Min-max	3 – 78
Non-nosocomial isolates	35/141 (24.8)
Non-nosocomial isolates from patients whom were hospitalized during previous 1 year	17/35 (48.6)
Colonizer	75/141 (53.2)
Significant isolate	66/141(46.8)
Prior antibiotics use	93/141
1 course of antibiotic	37 (39.8)
2 courses of antibiotics	30 (32.3)
3 and more courses of antibiotics	26 (28.0)
Quantitative variables, i.e. age, duration of stay in hospital, duration of stay in ICU, number of surgery are presented as median (interquartile range) because they do not have a normal distribution; categorical variables are presented as number (%).	

**Table-III:** Antimicrobial susceptibility of *Acinetobacter *spp. isolated from 141 patients

*Antibiotics*	*Acinetobacter spp. Isolates*		
	*No. Resistance (%)*	*No. Intermediate (%)*	*No. Sensitive (%)*
Ampicillin	134 (95)	3 (2.1)	4 (2.9)
Cefuroxime-paranteral	114 (80.8)	17 (12.1)	10 (7.1)
Cefotaxime	110 (78)	27 (19.2)	4 (2.8)
Amoxicillin-clavulanate	107 (75.9)	14 (9.9)	20 (14.2)
Ciprofloxacin	104 (73.8)	2 (1.4)	35 (24.8)
Meropenem	103 (73)	1 (0.7)	37 (26.3)
Ceftazidime	103 (73)	1 (0.7)	37 (26.3)
Imipenem	102 (72.3)	-	39 (27.7)
Piperacillin tazobactam	101 (72.7)	6 (4.3)	32 (23)
Ampicillin sulbactam	100 (70.9)	-	41 (29.1)
Gentamicin	99 (70.2)	4 (2.8)	38 (27)
Cefepime	95 (73.1)	1 (0.7)	34 (26.2)
Cefoperazone sulbactam	92 (65.2)	8 (5.7)	41 (29.1)
Netilmicin	88 (62.4)	3 (2.1)	50 (35.5)
Amikacin	82 (62.7)	3 (2.1)	46 (35.2)
Polymyxin B	-	-	28 (100)
Tigecycline (for 121 samples)	9 (7.4)	27 (22.3)	85 (70.3)

## DISCUSSION

In the past two decades, *Acinetobacter *spp. have been considered as important opportunistic pathogens responsible for nosocomial infections, especially among patients in intensive care units (ICUs).^5^ The data in this study showed that most of these isolates were obtained from soft tissue and wound followed by respiratory tract. Most of them were isolated from the intensive care unit (ICU), which suggests that seriously ill patients in ICUs have a greater chance of becoming colonized or infected by *Acinetobacter* spp. especially through the soft tissue/wound and respiratory tract. Similarly Falagas et al^6^ reported that infections caused by *Acinetobacter* spp. are more common in the ICUs in Asian and European hospitals and are lower in the United States hospitals, also Carlet et al^7^ and Peleg et al^8^ mentioned the prevalence of hospital-acquired infections could be as high as 25% in an ICU and there is a problem with nosocomial infection which is only one third of hospital-acquired infections that are avoidable.

Our data show that the median age of patients is 54 (31-66) which indicates the infection by *Acinetobacter *spp. occurs in elderly patients. One study documented the distribution of MDR-*Acinetobacter* was greatest in the >65 age group and long term care facilities.^9^ Also, duration of stay in hospital effected to acquire *Acinetobacter *infections, as in our data point out that this duration is from 3 days to 78 days. In several studies which examined nosocomial, blood stream, and burn infections explained antibiotic-resistance *Acinetobacter* infections are associated with longer hospital stays.^10^^,^^11^

The present study showed that most patients (75.2%) exposed to *Acinetobacter infection *after 48 hours during hospitalization are considered as nosocomial patients. Reports of Chang et al^12^ and Enoch et al^13^ mentioned that* Acinetobacter* spp. emerged as a crucial pathogen in health care - associated and nosocomial infections with high mortality, and was difficult to treat efficiently. Munoz-Price^5^ also reported that hospital acquired *Acinetobacter *is often multidrug resistance and widespread.

In the present study, the isolates were predominantly colonizers (53.2%). Thus, *Acinetobacter *spp. colonization of the hospital environmental may lead to infection because they survive on both moist and dry surfaces for long periods in the hospital environment.^14^ This feature of *Acinetobacter* is helpful to survive in hospital environments and cause infection and eliminating *Acinetobacter* spp. from clinical materials is difficult.^15^

In this study, the frequency of patients who received antibiotics before the isolation of *Acinetobacter* spp. is 66.0%. The most common antibiotics that patients received before the diagnosis of *Acinetobacter *spp. were meropenem (22.5%), followed by ceftriaxone (17.6%), piperacillin-tazobactam (13.4%). Other studies^16^^,^^17^ have reported that prior exposure to antibiotics as one of the risk factors for acquisition of *Acinetobacter* infections. Besides prior exposure to antibiotics^18^, other factors include long stay in hospital^19^, ICU admission, using tubes and catheter^18^ and furthermore, transmission between colonized or infected patients directly from hospital equipment or through the hands of health care workers, were also reported as risk factors for acquisition of *Acinetobacter* spp.^20^

In general, treatment options for *Acinetobacter *infections are limited and there are not any controlled trials to show therapeutic choices. Also, carbapenems and colistin are the options of choice for the most drug-resistant infections.^1^ In view of increasing resistance of *Acinetobacter* to carbapenem, polymyxins have been considered an option for the treatment of multidrug resistant *Acinetobacter* spp. infections.^6^

 In our study, except for polymyxin B, tigecycline had high sensitivity rates to *Acinetobacter* isolates. Therefore, this new glycylcycline agent has bacteriostatic activity against multidrug Acinetobacter spp. to is the appropriate agent for skin and soft tissue infection caused by MDR Acinetobacter spp.^23^ However; there are some reports of high level resistance to tigecycline among MDR *Acinetobacter* spp.^23^

In our study, except for polymyxin B, tigecycline had high sensitivity rates to *Acinetobacter* isolates. Therefore, this new glycylcycline agent has bacteriostatic activity against multidrug *Acinetobacter* spp.^23^ However; there are some reports of high level resistance to tigecycline among MDR *Acinetobacter* spp.^24^

In conclusion, the present study provided some information about the patients that are prone to *Acinetobacter* infections based on their clinic-epidemiological features. It also showed that there were high resistant rates of *Acinetobacter* isolates to common antibiotics except for polymyxin B which becomes emerging problem in combating nosocomial infections in Malaysia. The current findings might be helpful to strategize infection control measures and guidance for prudent use of antibiotic against *Acinetobacter* infections. 
